# Molecular determinants associated with temporal succession of SARS-CoV-2 variants in Uttar Pradesh, India

**DOI:** 10.3389/fmicb.2023.986729

**Published:** 2023-02-01

**Authors:** Smita Pal, Poonam Mehta, Ankita Pandey, Anam Ara, Ujjala Ghoshal, Uday C. Ghoshal, Rajesh Pandey, Raj Kamal Tripathi, Prem N. Yadav, Ramachandran Ravishankar, Tapas K. Kundu, Singh Rajender

**Affiliations:** ^1^CSIR-Central Drug Research Institute, Lucknow (CSIR-CDRI), Lucknow, India; ^2^Academy of Scientific and Innovative Research (AcSIR), Ghaziabad, India; ^3^Department of Microbiology, Sanjay Gandhi Post Graduate Institute of Medical Sciences, Lucknow, India; ^4^Department of Gastroenterology, Sanjay Gandhi Post Graduate Institute of Medical Sciences, Lucknow, India; ^5^CSIR-Institute of Genomics and Integrative Biology, New Delhi, India; ^6^Jawaharlal Nehru Centre for Advanced Scientific Research (JNCASR), Bangalore, India

**Keywords:** SARS-CoV-2 genome, COVID-19, vaccination breach, spike mutations, Delta variant, omicron variant, COVID-19 waves

## Abstract

The emergence and rapid evolution of Severe Acute Respiratory Syndrome Coronavirus 2 (SARS-CoV-2) caused a global crisis that required a detailed characterization of the dynamics of mutational pattern of the viral genome for comprehending its epidemiology, pathogenesis and containment. We investigated the molecular evolution of the SASR-CoV-2 genome during the first, second and third waves of COVID-19 in Uttar Pradesh, India. Nanopore sequencing of the SARS-CoV-2 genome was undertaken in 544 confirmed cases of COVID-19, which included vaccinated and unvaccinated individuals. In the first wave (unvaccinated population), the 20A clade (56.32%) was superior that was replaced by 21A Delta in the second wave, which was more often seen in vaccinated individuals in comparison to unvaccinated (75.84% versus 16.17%, respectively). Subsequently, 21A delta got outcompeted by Omicron (71.8%), especially the 21L variant, in the third wave. We noticed that Q677H appeared in 20A Alpha and stayed up to Delta, D614G appeared in 20A Alpha and stayed in Delta and Omicron variants (got fixed), and several other mutations appeared in Delta and stayed in Omicron. A cross-sectional analysis of the vaccinated and unvaccinated individuals during the second wave revealed signature combinations of E156G, F157Del, L452R, T478K, D614G mutations in the Spike protein that might have facilitated vaccination breach in India. Interestingly, some of these mutation combinations were carried forward from Delta to Omicron. *In silico* protein docking showed that Omicron had a higher binding affinity with the host ACE2 receptor, resulting in enhanced infectivity of Omicron over the Delta variant. This work has identified the combinations of key mutations causing vaccination breach in India and provided insights into the change of [virus’s] binding affinity with evolution, resulting in more virulence in Delta and more infectivity in Omicron variants of SARS-CoV-2. Our findings will help in understanding the COVID-19 disease biology and guide further surveillance of the SARS-CoV-2 genome to facilitate the development of vaccines with better efficacies.

## 1. Introduction

Comprehending the unremitting molecular evolution of SARS-CoV-2 genome is essential to control the devastating surge of the COVID-19 pandemic. The pace of research has to match the pace of SARS-CoV-2 evolution to tackle the spread of virus. Accelerated research and concrete efforts worldwide resulted in the development of vaccines to combat the pandemic; nevertheless, new variants have rendered vaccines ineffective at some points (Dubey et al., 2022). Emerging variants, being facilitated by new signature mutations, have rapidly outcompeted the prior circulating variants (MacLean et al., 2021; Purushotham et al., 2021). In India, the first surge of COVID-19 gained its momentum in March 2020, which declined in the late July 2020. Later, in March 2021, the noxious variant Delta broke out in India for the first time and conquered the prior circulating variants to dominate the second wave (Gupta, 2021; Jha et al., 2021). In late November 2021, the first Omicron variant was detected in South Africa, which after its first detection in India in December 2021, gradually replaced Delta. Interestingly, although Delta is more virulent in terms of COVID-19 disease severity, Omicron replaced Delta by acquiring increased transmissibility, generating neutralizing antibodies and mutational fitness over natural selection pressure (Singh and Yi, 2021; Petersen et al., 2022).

COVID-19 vaccination in India started in mid-January, 2021. The vaccine was offered free of cost by the Government of India in staggered phases at various centers across the country, starting with the frontline and healthcare workers. This was followed by the next phase of COVID-19 vaccination to the elderly population starting March, 2021. Vaccination to the general population was opened in May 2021 (Purohit et al., 2022). In the beginning, people did not actively take vaccine despite efforts from the government. Apart from significant mortality in the first and the second waves in the elderly population, the second wave of COVID-19 resulted in a much higher death rate in below 45 years age group in comparison to the first wave (Purohit et al., 2022). The breakout of Delta variant with a very high morbidity and mortality rate forced people to actively seek COVID-19 vaccination (Vishvkarma and Rajender, 2020). Eventually, vaccination was in full swing in the months of June–July, 2021. However, only one fourth of Indian population had received the first dose of vaccine and only 6% of population had received both the doses by July 2021 (Choudhary et al., 2021). However, In Uttar Pradesh, 13% of the population had received at least one dose and 3% had completed two doses by July 2021.

After its first appearance in China in the late 2019, the virus has continuously evolved by either substitutions or deletions, resulting in significant and unexpected changes in its virulence and infectivity (Abraham, 2021). Particularly, mutations in the Spike protein have driven this evolution and have caught attention (Banerjee et al., 2021; Chan and Zhan, 2022). Some of these mutational events have driven regional spread the virus, causing havoc in closed territories, certain countries, or throughout the world (Rochman et al., 2021; Williams and Burgers, 2021). Among factors that could affect COVID-19 presentation, severity and eventual outcome, viral genome variations remain one of the most prominent and interesting factors. In order to understand the molecular determinants associated with mutation-driven evolution, we sequenced SARS-CoV-2 genomes from the first, second and third wave of COVID-19 in Uttar Pradesh, India.

## 2. Material and methodology

### 2.1. Sample collection

COVID-19 research was approved by the Institutional Biosafety Committee of the Central Drug Research Institute, Lucknow and the Institutional Ethics Committee of Sanjay Gandhi Postgraduate Institute of Medical Sciences (SGPGI), Lucknow. The samples for this study were collected from Sanjay Gandhi Post Graduate Institute of Medical Sciences (SGPGIMS), Lucknow and the COVID-19 testing facility of the Central Drug Research Institute (CDRI), Lucknow. SGPGI is the largest state hospital in Uttar Pradesh and served as the biggest COVID-19 facility during the pandemic. The hospital received COVID-19 samples from the patients visiting the COVID-19 clinic for diagnosis, treatment or emergency care. The COVID-19 testing facility of CDRI is a government approved facility for testing of samples collected by various government approved centers for COVID-19 surveillance. The facility during its peak operation received 1,000 samples per day. Both of these facilities received patients or samples from Uttar Pradesh only. A total of 544 RNA samples from confirmed COVID-19 cases arising from different urban and rural areas of Uttar Pradesh (2020–2022), India, were subjected to Oxford Nanopore Technology (ONT) sequencing and clade analysis.

During the first wave (May 2020 to August 2020), 87 RNA samples having RT-PCR Ct value <30 were collected from different places of Lucknow and Jhansi. This sample cohort consisted of 78.65% male and 21.83% female patients.

During the second wave (April 2021–July 2021), 218 RNA samples with RT-PCR Ct value <30 patients were collected. The samples were from different cities of Uttar Pradesh, including Lucknow, Jhansi, Lalitpur, Ayodhya, and Orai. The age of the patients ranged from 12 years to 80 years, consisting of 87% males and 13% females. This cohort consisted of 64% fully vaccinated and 46% partially vaccinated (completed only the first dose) and 18% unvaccinated individuals. In total population, 24% were asymptomatic and rest 76% had symptoms like cold fever, body pain, breathing problem, abdominal disturbances.

During the third surge of COVID-19 in Uttar Pradesh (December 2021–January 2022), 239 samples were collected from symptomatic individuals from different districts, which consisted of 59.8% males and 40.2% females, and the entire group was vaccinated. The patient age ranged from 11 to 75 years.

### 2.2. SARS-CoV-2 genome sequencing

#### 2.2.1. Library preparation

RNA samples were amplified by using primers of ARTIC nCov-2019 (version 3). Briefly, the RNA template was converted into complementary DNA (cDNA) using the high capacity cDNA reverse transcription kit (Applied Biosystems, United States) by keeping the sample in a thermocycler initially for 10 min at 25°C, followed by 60 min at 37°C, again 60 min at 37°C and finally 5 min at 85°C. The second run of PCR was performed by using AmpliTaq Gold™ 360 Master Mix while exposing the cDNA samples initially at 95°C for 10 min, followed by 35 cycles at 95°C for 45 s, 59°C for 5 min and 72°C for 45 s, followed by a final incubation at 72°C for 7 min. The expected PCR product size was 450 bp. The samples showing good quality bands on 1.8% agarose gel were considered for downstream library preparation.

Sequencing of the Spike region was targeted for sequencing in maximum number of the cases. The libraries for the first 120 samples were prepared by Oxford Nanopore native barcode kit (NBD104 and EXP-NBD114), where samples were first cleaned (by AMPureXP beads) after performing end prep, and the barcodes were ligated. After barcoding, all the samples were pooled in a single Eppendorf tube and adapter ligation was carried out at room temperature and the final washing was done. The libraries for the rest of the samples were prepared using Oxford Nanopore rapid barcode 96 kit (SQK-RBK110.96). Briefly, the samples were prepared by first ligating them in the barcode plate and then pooling all the samples together for magnetic bead wash. Adapters were ligated at room temperature before priming the flow cell.

#### 2.2.2. Flow cell priming and sequencing on MinION

For Nanopore MinION sequencing, spot-on flow cells of R9 version were used (FLO-MIN106D). The flow cells were primed according to the manufacturer’s instructions, using flush buffer and flush tether. 800-1,000 ng of the library was premixed with sequencing buffer and loading beads just prior to loading on spot on port of the flow cell. The base quality filter cut off value of 8 was used for accurate base calling. The sequencing was continued upto >900 Mb for a batch of 96 samples to generate approximately 300X coverage, giving nearly 25,000–30,000 reads per sample.

#### 2.2.3. Post sequencing read filtering and functional annotation

After completion of the sequencing process, barcoded reads were analyzed by ARTIC nCoV pipeline.^1^ Briefly, the ARTIC environment was created first and then the reads having 400–600 bp length were filtered from unwanted reads using guppy commands. Consensus sequences were made from the amplicons by nanopolish and subjected to EPI2ME to check the coverage quality. The final sequences were submitted to annotate the ORFs in VIGOR_4_ (Viral Genome ORF Reader; Wang et al., 2010).

### 2.3. Clade analysis and mutation tracking

The trimmed sequences were checked in Pango, Nextclade and GISAID-CoVsurver mutation app for clade and lineage characterization. GISAID (Khare et al., 2021) employs EpiCoV database to assign phylogenetic clades and lineages to the sequences. Nextclade works by identifying the differences between the query sequence and the original SARS-CoV-2 Wuhan sequence to identify matches and mutations and characterizes the clades.

### 2.4. Neighborhood homology mapping and phylogeny

The circular representations of homologous comparison of SARS-CoV-2 Spike sequences from different time frames (consensus of the highest abundant variants from the 1^st^, 2^nd^ and 3^rd^ waves) were conducted by MUSCLE2 (Edgar, 2004) and Proksee (Grant and Stothard, 2008),^2^ with visualization of GC skewness, ORF distribution, annotation and blast comparison. Phylogeny tree was constructed and visualized by MEGA 11 (Tamura et al., 2021), based on the maximum likelihood, followed by heat map plotting of Pearson coefficient of mean distance matrix between amino acid placements.

### 2.5. *In silico* docking with ACE2 receptor and binding free energy calculation

The consensus genome sequences, covering all major mutations with the highest quality score, were selected as representative genomes of the second and third waves. The pdb files of Spike trimeric glycoproteins from the ViGor annotated file were created by Phyre2. Pdb file was also generated for angiotensin converting enzyme 2 (ACE2, NCBI Gene ID: 59272), which is the universal receptor for human coronavirus HCoV-NL63 and severe acute respiratory syndrome coronaviruses (SARS-CoV) and SARS-CoV-2. *In silico* docking was performed by HADDOCK 2.4 (Roel-Touris et al., 2019),^3^ Spoton (Moreira et al., 2017) and Hawkdock server (Weng et al., 2019).^4^ The highest score model was selected from the top 10 solutions provided by the servers. The binding free energy in terms of Gibb’s free energy (−ΔG) and dissociation constant K_d_ were calculated by PRODIGY (Xue et al., 2016).^5^ For visualization, PyMOL platform was used and all.pdb files were checked for model authenticity, Z score and Ramachandran plot stability by ProSA, ProQ, and PdbSum (Laskowski et al., 2018).

## 3. Results

**Figure 1:**
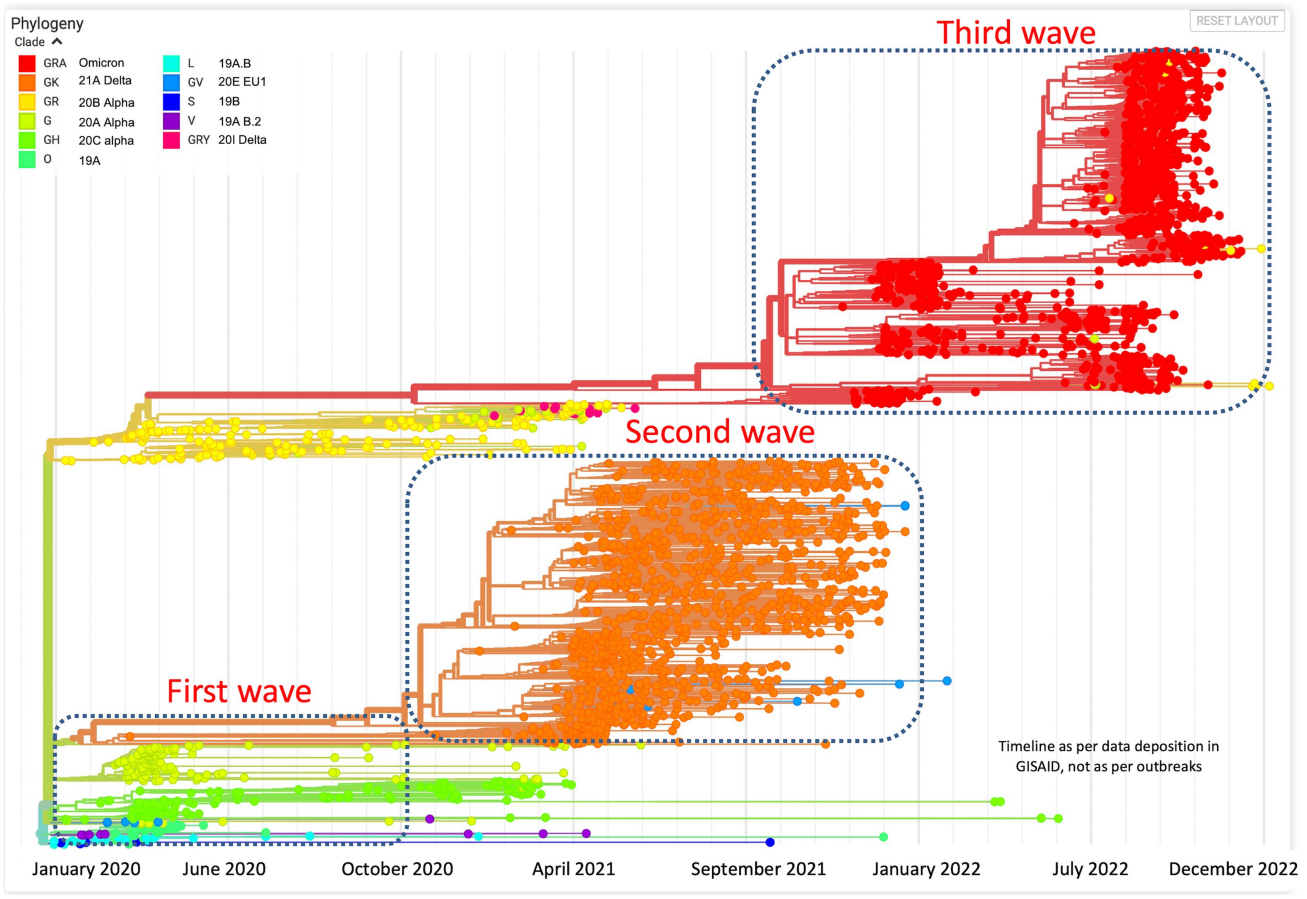
Phylogenetic analysis of SARS-CoV-2 variants in India during the period of January 2020–December 2022.

**Figure 2:**
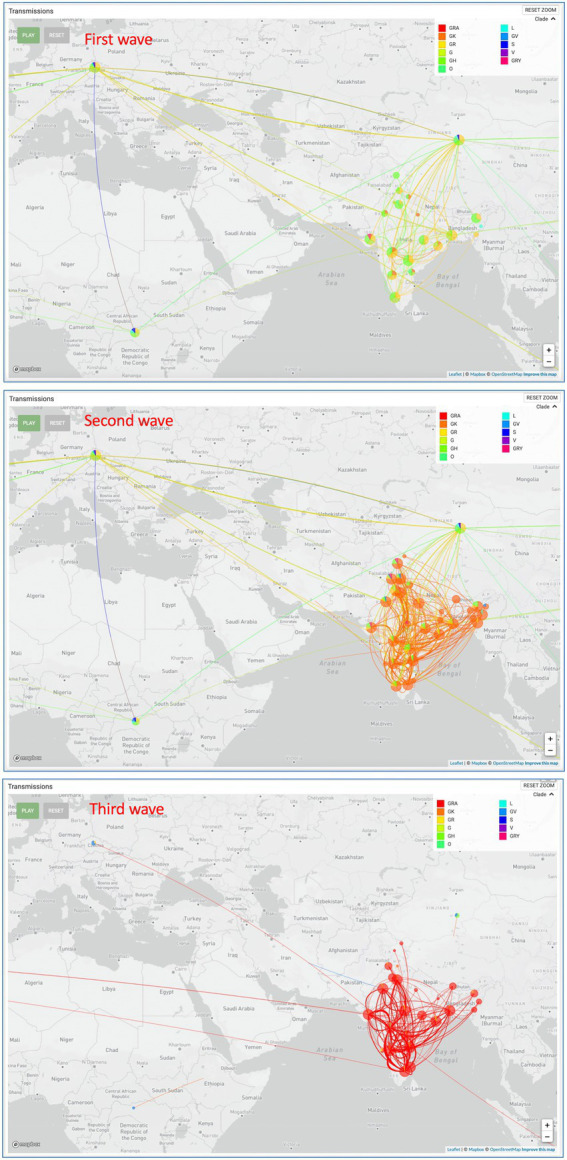
SARS-CoV-2 variant distribution and transmission data for the first, second and third waves.

All the sequences are publicly available in the GISAID database under Asia/India/Uttar Pradesh/subhead CDRI submission. The spike gene region was covered in all the cases, spike region with other regions was covered in 20% of the cases and complete genome coverage was achieved in 36% of the cases. However, we have largely focused on the Spike region only. For a comparative account, the phylogenetic analysis of SARS-CoV-2 variants in India during the period of January 2020–December 2022 is presented in Figure 1 with variant distribution and transmission data corresponding to the first, second and third waves presented in Figure 2.

### 3.1. Evolution of SARS-CoV-2 clades

#### 3.1.1. First wave

**Figure 3:**
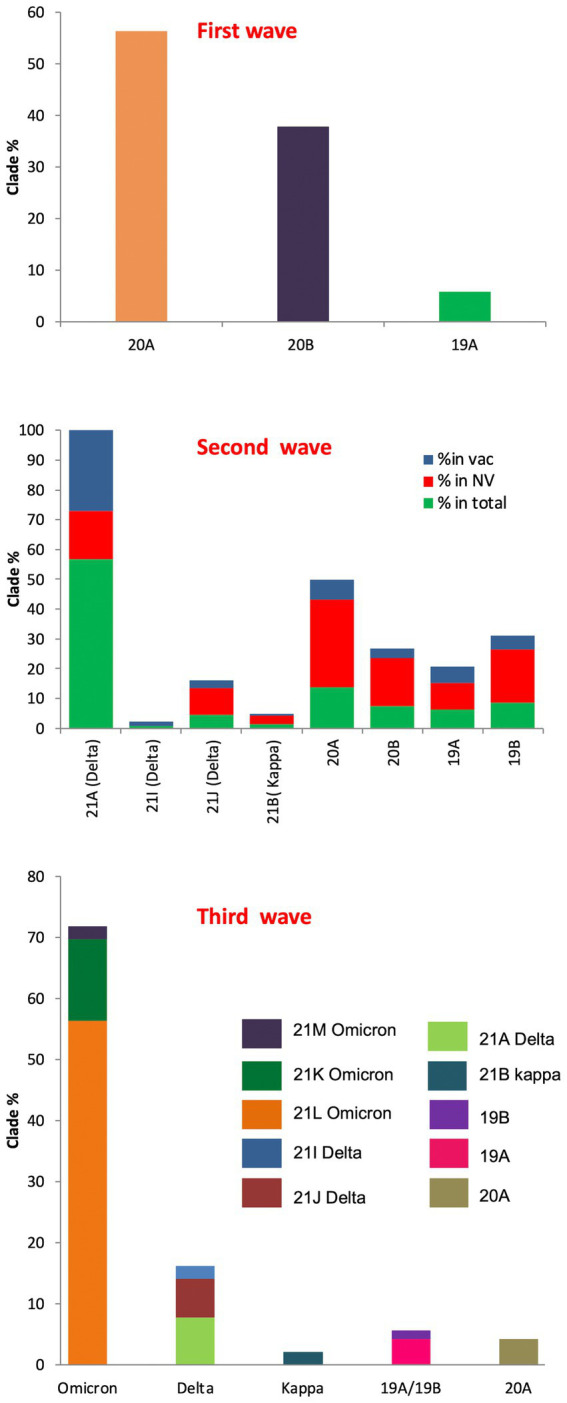
SARS-CoV-2 clade profile distribution during COVID-19 first to third waves in Uttar Pradesh, India.

In the first wave, 56.32% of the samples were found to have 20A Alpha, followed by 37.93% with 20B and 5.74% with 19A, presenting 20A Alpha to be the most dominant clade in Uttar Pradesh (Figure 3). Since no vaccine was available at that time, these samples were not classified according to the vaccination status.

#### 3.1.2. Second wave

During the second wave, the vaccination drive was in full swing in India and this provided us with the opportunity to classify the samples into vaccinated and unvaccinated groups. The overall prevalence of 21A Delta in Uttar Pradesh at this time was 56.88%, with a relatively much higher frequency of 21A Delta (75.84%) in vaccinated people. The unvaccinated pool majorly carried 20A (29.41%), 20B clade (16.17%) and 21A Delta (16.17%) variants. Other delta variants (21 J + 21I) were more common in unvaccinated individuals (8.83%) in comparison to vaccinated individuals (3.96%; Figure 3; Table 1).

**Table 1:** The comparison of frequencies of the Spike mutations between vaccinated and unvaccinated COVID-19 patients during the second wave.

Spike mutation	Unvaccinated (%)	Vaccinated (%)	Fisher exact *p* value
Spike_R158del	17.86	96.92	6.84e-15
Spike_T478K	13.79	94.32	1.32e-16
Spike_D614G	100	95.23	0.179
Spike_E156G	19.23	78.75	8.03e-8
Spike_D950N	13.33	87.18	6.36e-7
Spike_L452R	12.50	66.30	1.11e-8
Spike_F157del	15.63	9.09	0.328
Spike_P681R	36.36	62.16	0.049
Spike_E484Q	2.78	Not detected	0.281
Spike_H1101D	5.56	Not detected	0.068
Spike_S982A	2.38	Not detected	0.280
Spike V1104L	Not detected	1.00	1.00

#### 3.1.3. Third wave

The situation took a turn when Omicron started replacing other variants in the mid of December 2021 in Uttar Pradesh. The cumulative percentage of Omicron was found to be 71.8%, which outcompeted Delta (16.19%) by the end of January 2022. Although the ratio of the sister lineages of 21 K and 21 L of Omicron differed from state to state, 21 L Omicron was found to be dominant (78.43%) over 21 K sub lineage (18.62%) in Uttar Pradesh. Interestingly, the frequency of 21 K Omicron was comparable to the frequency of Delta variant during this period (Figure 3).

### 3.2. Evolution of SARS-CoV-2 genome mutations

#### 3.2.1. First wave

During the first wave, 20A Alpha carried the Spike protein mutations I285S and D614G. Other mutations were Q168H, M169V, S171stop, D172H, L174stop and N176S in NSP14; Q57H and G254stop in NS3; I199L, I210V, M211L, L212S, V213I, Y214del, C215A and F216L in NSP6; and L27F mutation in Envelop E.

20B carried D614G and Q677H mutations in the Spike protein. Other mutations included I124V, V149F in NSP6; R203K, G204R, D371V in Nucleocapsid N, K412N in NSP3, P323L in the NSP12 protein, L21F in the Envelop E protein.

**Figure 4:**
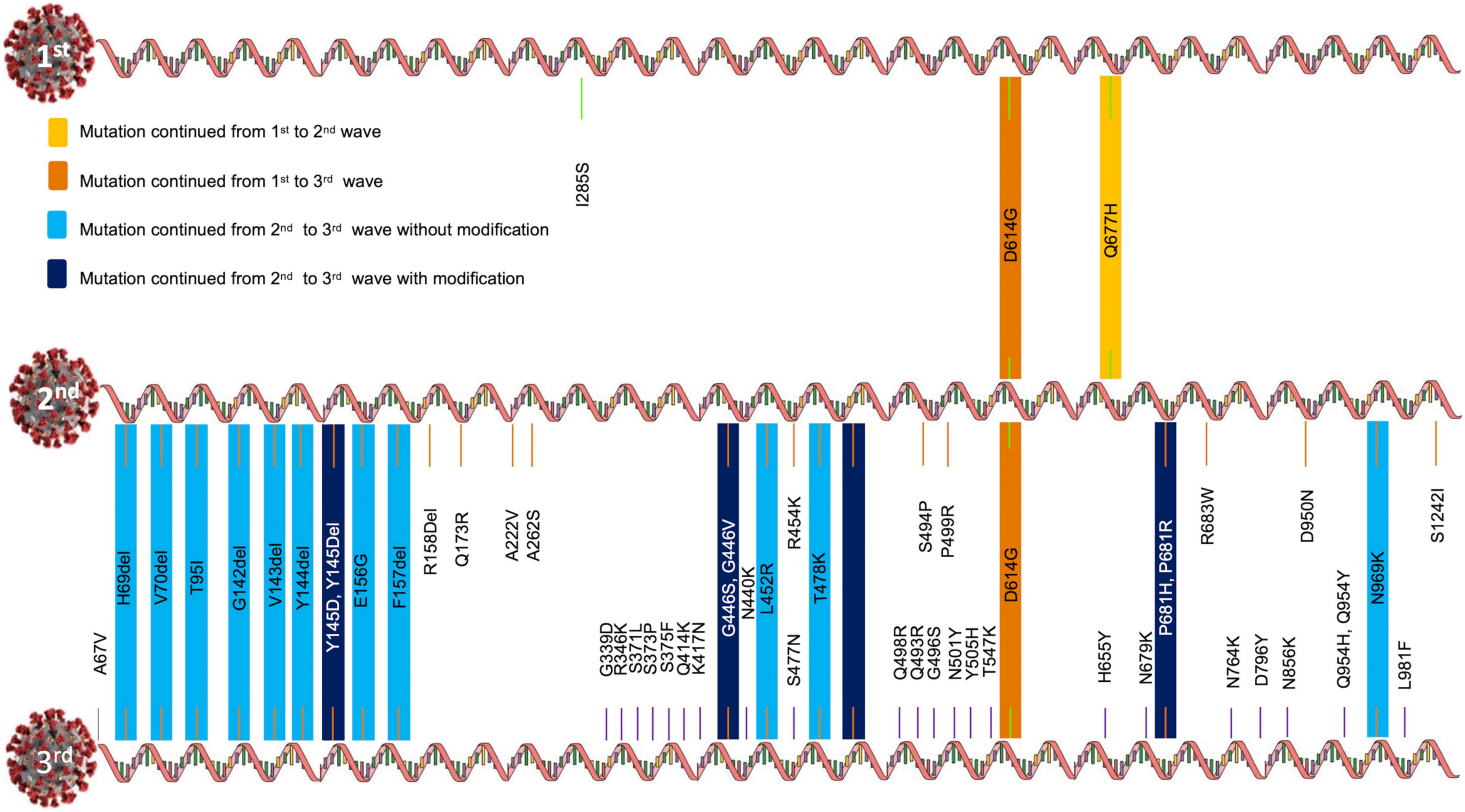
Evolution of Spike mutations during COVID-19 first to third waves in Uttar Pradesh, India.

Similarly, the 19A clade carried I285S, D614G and Q677H in the Spike protein. Other mutations included A185V, V381A in NSP12 and V1762F in NSP3 proteins. Interestingly, out of the three Spike mutations, only D614G and Q677H were carried forward to the second wave and only D614G was passed to the third wave (Figure 4).

#### 3.2.2. Second wave

Nextclade and GISAID majorly annotated G142D, V143del, Y144del, Y145del, E156G, F157del, R158del, Q173R, A222V, A262S, Q414K, G446V, L452R, R454K, T478K, E484Q, S494P, P499R, D614G, Q677H, P681R, R683W, D950N, S1242I mutations in the Spike glycoprotein of 21A Delta.

A67V, H69del, V70del, T95I, G142D, V143del, Y144del, Y145del, E156G, F157del, R158G, R158del, L452R, T478K, E484Q, P499R, D614G, P681R, D950N, Q954Y, N969K, V1104L mutations were present in the Spike proteins of 21 J Delta.

A67V, H69del, V70del, G142D, L452R, T478K, E484Q, D614G, P681R, D950N mutations were present in the Spike protein of 21I Delta variant.

A number of Spike mutations were passed to the Omicron variant without modifications and a few were passed to the Omicron variant with modifications (Figure 4). Omicron shared at least one Spike mutation (D614G) with Delta and 19A and 20A variants.

#### 3.2.3. Third wave

By the time the third wave arrived (early December 2021 to February 2022), the majority of the population was vaccinated; hence, we could not classify these samples as per their vaccination status. However, a number of spike mutations, such as A67V, H69del, V70del, T95I, G142del, V143del, Y144del, Y145D, G339D, R346K, S371L, S373P, S375F, K417N, N440K, G446S, S477N, T478K, E484A, Q493R, G496S, Q498R, N501Y, Y505H, T547K, D614G, H655Y, N679K, P681H, N764K, D796Y, N856K, Q954H, N969K, L981F, were found in the Omicron sister lineages 21 K and 21 L (Figure 4). Interestingly, the key determinant mutations for vaccination breach (F157del, R158del, L452R, T478K, D614G, P681R) were present in the Omicron variant as well. Moreover, some mutations, earlier detected in Delta, were found in different forms in Omicron (Q954Y → Q954H and E484Q → E484A, Figure 4). Essentially, Omicron contains profoundly higher number of mutations, yet some mutations were lost in the evolution from Delta to Omicron (R158G, A262S, S494P, S1242I).

### 3.3. Unique mutation combinations in delta may be responsible for vaccination breach

The frequencies of Spike protein mutations, such as E156G, R158del, L452R, T478K, and D950N were significantly higher in the vaccinated population in comparison to unvaccinated individuals (Table 1).

**Figure 5:**
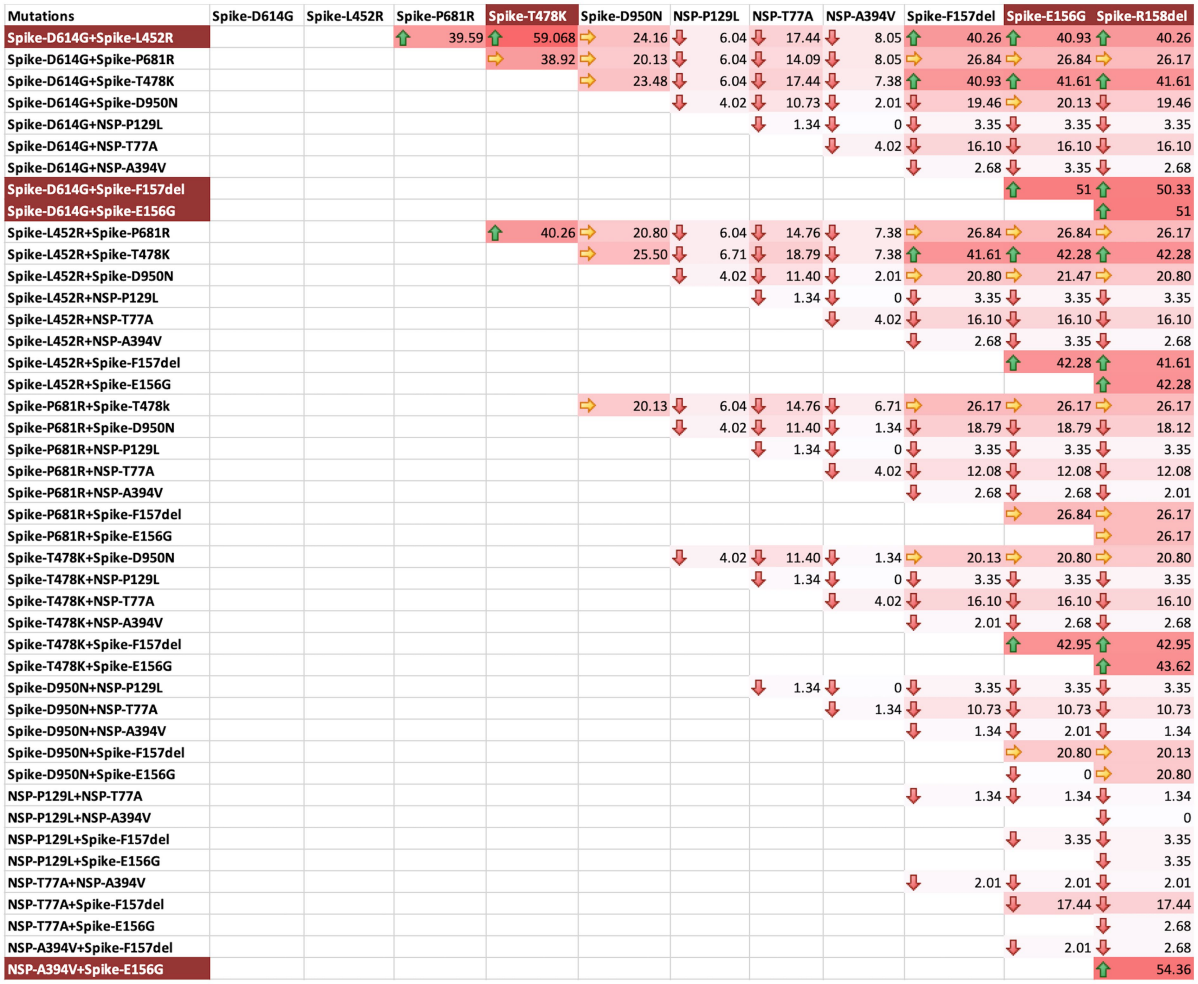
Three mutations combination matrix for identification of mutations facilitating vaccination breach during COVID-19 s waveThree mutations combination matrix for the identification of mutations facilitating vaccination breach during COVID-19 second wave. The background colour gradient from white to red indicates increasing frequency of combinations. Green arrows represent high frequency (>40), yellow arrows represent moderate frequency (20-40), and red arrows represent low frequency (<20). The background highlight in the mutation names indicates the most frequent combinations.

We also asked if there were specific combinations of mutations that resulted in frequent vaccination breach during the second wave. For this, a matrix analysis of three Spike mutations at a time was undertaken. L452R, T478K, and D614G combination was found to be the most frequent combination, followed by E156G, F157Del and D614G combination, E156G and R158Del and D614G combination, F157Del, R158Del and D614G combination, F157Del, L452R and D614G combination, and E156G, L452R and D614G combination in the vaccinated people (Figure 5). These combinations highlight E156G, 157Del, L452R and D614G as the most significant mutations for vaccination breach. Though only Spike protein variations have been emphasized, we also observed a combination of NSP mutations with two Spike mutations to be very frequent among vaccinated individuals (Figure 5). LINK Excel.Sheet.12 “E:\\CDRI 2021-22\\covid19\\PAPER\\manuscript\\THREE_COMBINATIONS_RESULTS_1.xlsx” Sheet1!R3C3:R47C14 \a \f 5 \h \* MERGEFORMAT

### 3.4. SARS-CoV-2 binding affinity improved during evolution

**Figure 6:**
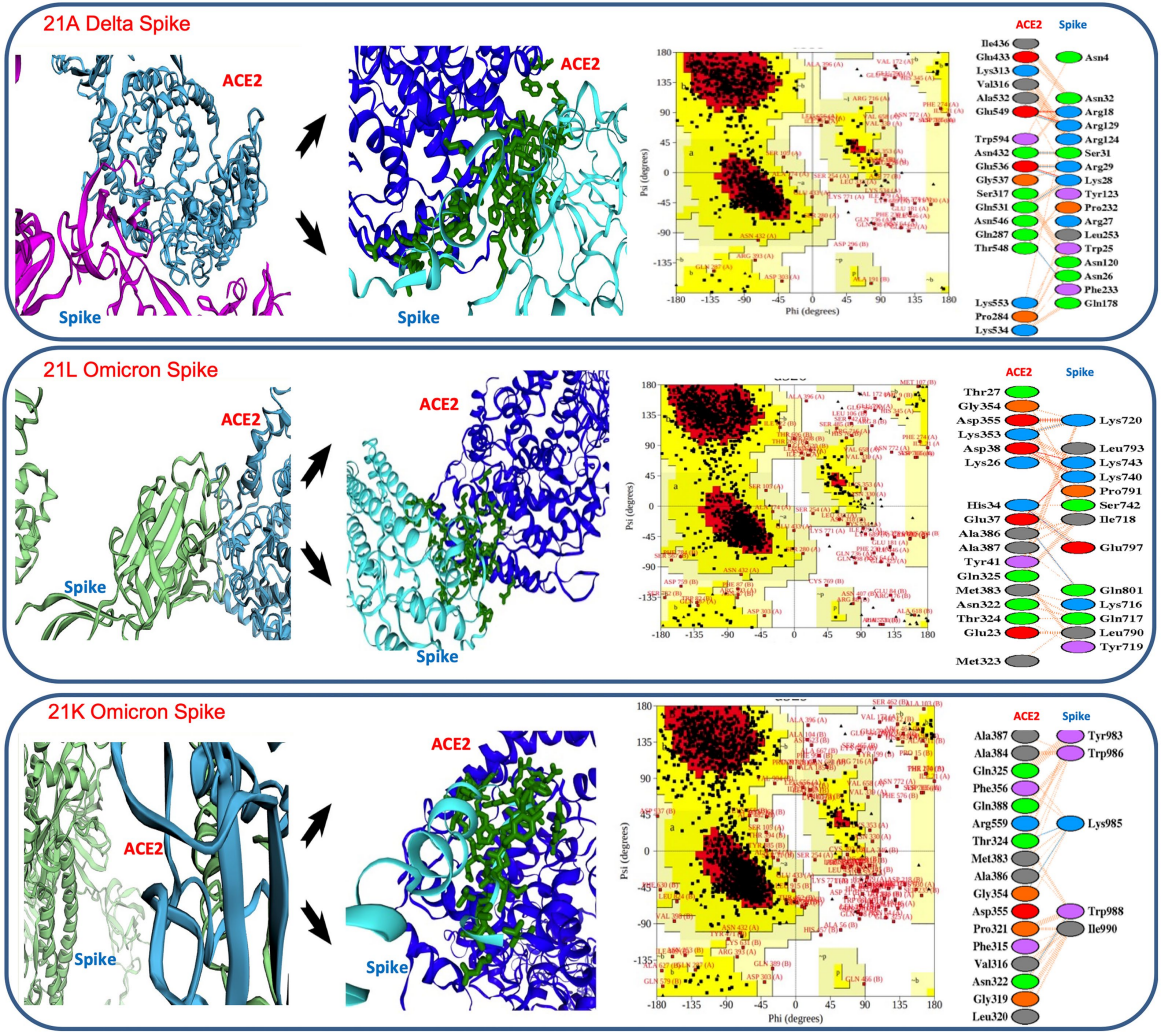
Details of *in silico* docking of SARS-CoV-2 Spike protein with host ACE2 receptor (leftmost across all three panels), intrinsic view of docking area (middle of all three panels), Ramachandran plotting indicating the stability of the docked complex (third block across all three panels), details of the amino acids involved in docking (rightmost across all three panels). It shows 21 K Omicron Spike fused with ACE2 has the lowest stability as the major amino acids fall in the disallowed or restricted space.

The binding affinities and dissociation constants were considered as the key determinants of the pathogenesis and infectivity (Shang et al., 2020; Han et al., 2021; Supplementary Table S1). We evaluated if a change in the binding affinity between spike protein and human angiotensin-converting enzyme 2 (ACE2) receptor was responsible for a sudden shift in infectivity of SARS-CoV-2 in vaccinated cases. The overall configuration of the spike protein provides the ease of binding, as it contains many pores (>15 Å) and tunnels (>25 Å) near the binding cavity, facilitating a smooth entry. The calculation of binding affinity showed much higher affinity in Delta in comparison to the variants observed in the first wave. Similarly, Omicron showed a higher binding affinity and a lower dissociation constant than Delta (Figure 6).

In thermodynamic comparison (Supplementary Table S2), Delta consensus Pdb was found to involve 3 salt bridges, 6 hydrogen bonds and 125 non bonded contacts, offering binding free energy (BFE) of −20.2367 ± 1.118 kcal/mol while docking with ACE2. 21 L omicron- spike protein indulged 5 salt bridges, 3 hydrogen bonds and 103 non bonded contacts, contributing BFE of −61.8833 ± 2.254 kcal/mol, confirming a much higher affinity (Supplementary Table S2). Further, the lower dissociation constant (Kd) in 21 l Omicron spike-ACE2 complex (1.04E-07 ± 1.7E-07 kcal mol) than 21A Delta spike-ACE2 complex (7.75E-08 ± 7.19E-08 kcal mol) established the reason behind higher level spontaneous and more stable binding of the Omicron 21 L spike protein with the ACE2 receptor (Figure 6).

Later on, when 21 K Omicron variants were subjected to *in silico* docking with the ACE-2 receptor, it showed a higher Gibb’s free energy and a higher Kd value than Omicron 21 L as well as Delta, suggesting weaker binding, which may be the reason behind the lower number of 21 K Omicron cases in comparison to 21 L. Although the exact Kd values differ depending upon the tools and approaches of calculation, our values were quite similar with Shang et al. (2020) and Buratto et al. (2021). However, the gross lowering of dissociation constant as the SARS-CoV-2 evolved from 2020 to 2022 indicated the higher chances of infectivity as it evolved (Figure 6; Supplementary Table S2).

Moreover, when the receptor and spike complex was checked for configuration stability by Ramachandran plot, both Delta and 21 l Omicron showed similar percentages of allowed (98.3%) and disallowed regions (1.7%), whereas 21 K presented 2.6% amino acids falling in the disallowed region, which may result in lesser stability of the complex. Additionally, the number of proline residues was higher in the outer region of the spike protein in 21 K (Figure 6).

### 3.5. SARS-CoV-2 phylogeny and spike consensus homology mapping

**Figure 7:**
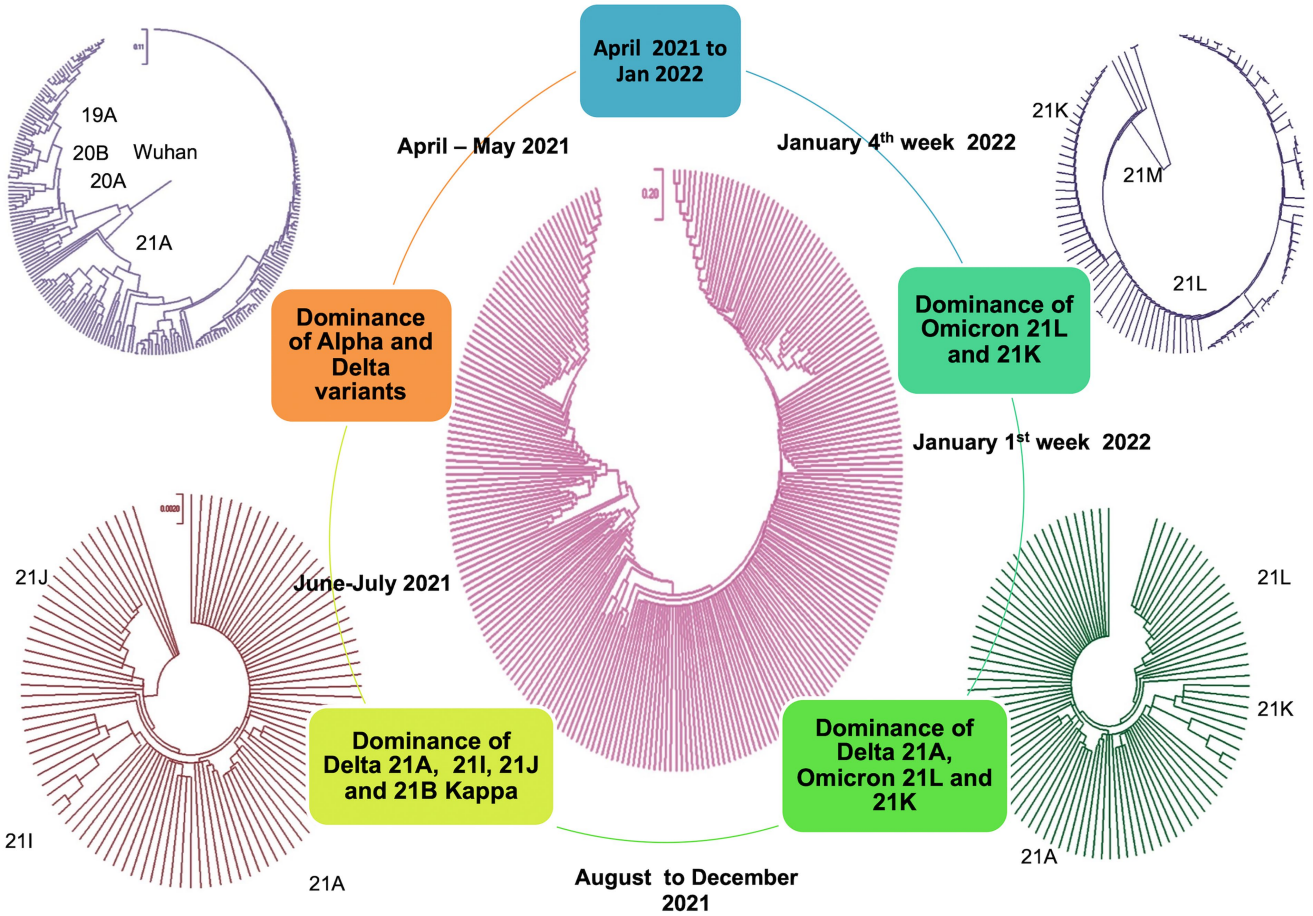
Molecular phylogenetic tree showing evolution distance profile during March 2021–January 2022 (2nd and 3rd waves).

MEGA11 was employed to find the molecular evolution from April 2021–June 2021 spread to December 2021–January 2022 spread. The nodes were elaborated further to capture the maximum likelihood homology in detail (Figure 7).

The tree topology indicated that the Delta lineages in Uttar Pradesh could arise from the ancestral lineage, but Omicron probably did not directly originate from any of the previous variants (Venkatakrishnan et al., 2021), instead it might have followed a cryptic genomic architecture involving a different recombination history (Bolze et al., 2022; Ou et al., 2022). The very long branch of the Omicron lineage in the time-calibrated tree might reflect less diversity within the group and a complex evolutionary history.

**Figure 8:**
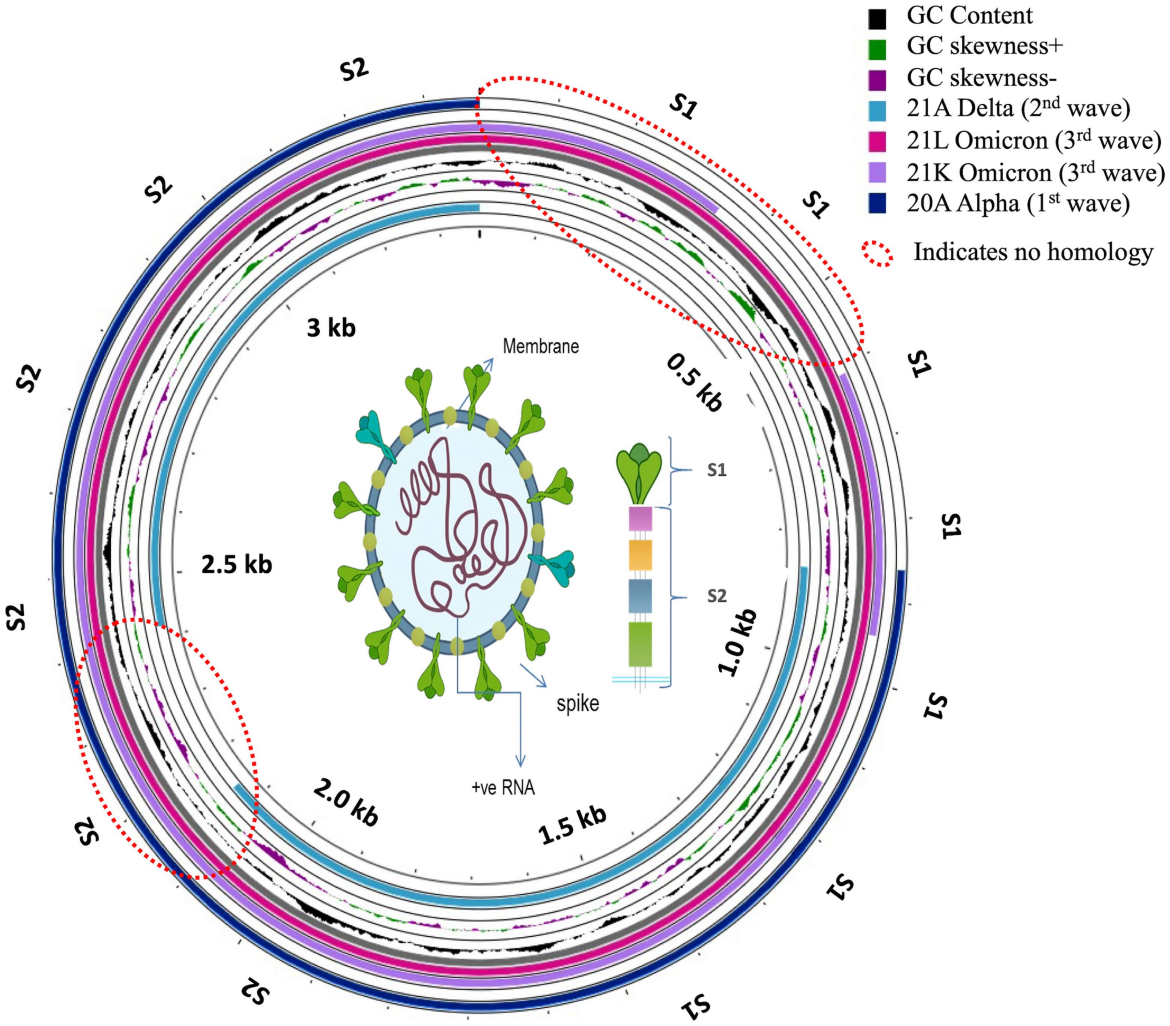
Circular presentation of neighborhood homology mapping of consensus Spike proteins for the first, second and third waves of COVID-19.

Proksee was used to find the neighborhood homology of Muscle.2 aligned spike sequences (Figure 8). The consensus (with the highest coverage) of the most abundant variant from each of the waves was classified by BLAST homology and mapped later in a circular presentation. The gap pattern or non-matched region reflected that although there was a significant homology in 20A Alpha and 21A Delta, suggesting a common parental root, Omicron did not share good homology with others, conferring the plausibility of mystic intervention in the evolutionary history (Thiruvengadam et al., 2022). The histogram pattern showed significant shifts in the curves due to several mutational changes, which abruptly shifted the GC skewness and made the domain a hot spot for upcoming events.

## 4. Discussion

### 4.1. SARS-CoV-2 genome evolved to cause breakthrough infections

Interestingly, India stood as an exclusive country where vaccination and the surge of second wave occurred concurrently, raising the selection pressure on the viral genome (Focosi and Maggi, 2022) and simultaneous immunity shift, antigenic drift, which might have triggered the origin and gradual rise of Delta in March 2021 (Kumar et al., 2020; Dhar et al., 2021; Gupta, 2021). COVID-19 vaccination in India started in mid-January, 2021. The vaccine was offered free of cost by the Government of India in phases at various centers across the country. Vaccination was in full swing in the months of June–July, 2021. Only one fourth of the population had received the first dose of vaccine and only 6% of population had received both the doses by July 2021 (Choudhary et al., 2021). The mortality in the first wave was significantly higher in the elderly population, but the second wave resulted in much higher mortality in less than 45 years age population in comparison to the first wave.

The first wave was dominated by 20A Alpha (56.32%) and 20B (37.93%), and 19A (5.74%). The second wave was dominated by 21A Delta (56.88%), with a relatively much higher frequency of 21A Delta (75.84%) in vaccinated people. The unvaccinated pool majorly carried 20A (29.41%), 20B clade (16.17%) and 21A Delta (16.17%) variants. Other delta variants (21 J + 21I) were more common in unvaccinated (8.83%) in comparison to vaccinated individuals (3.96%; Figure 3; Table 1). The third wave was dominated by Omicron (71.8%), which outcompeted Delta (16.19%) by the end of January (Purohit et al., 2022). Although the ratio of the sister lineages of 21 l and 21 K of Omicron differed from state to state, 21 l Omicron was found to be dominant (78.43%) over 21 K sub lineage (18.62%) in Uttar Pradesh. Some of the mutations that originated in the first and second waves were retained by the subsequent variants of the virus, suggesting their contribution to the fitness of the virus (Figure 4).

### 4.2. Specific mutation combinations facilitated vaccination breach

A key mutation noted in the first wave, D614G, significantly increased in frequency as the virus spread from Wuhan to Italy, United Kingdom and India (Mehta et al., 2021). D614G has higher dN/dS ratio (an indicator of selection pressure on coding genes; Volz et al., 2021). The emergence of other combinations of mutations further changed infectivity and virulence; however, D614G remained present and is still present in the Omicron variant as well. We observed that the combinations of spike mutations E156G, F157Del, L452R, T478K, D614G and grouping of NSP-A394V with spike E156G and R158Del evaded the protection provided by vaccination and brought the breakthrough infections in the second wave (Figure 5). Other studies from India also claimed that the emergence of L452R, T478K, E484Q, D614G and P681R mutations in the Spike protein was responsible for dynamic transmissibility and breakthrough of Delta variants in North, West and Mid India (Cherian et al., 2021; Joshi et al., 2021; Singh et al., 2021). The majority of these mutations were found to provide resistance by not being neutralized by convalescent sera and intrinsically enhancing the fusion of ACE-2 receptor with the S1 subunit of the trimeric spike glycoprotein (Kannan et al., 2022). Similarly, Wang et al. (2021), Pondé (2022) and others claimed that specific combinations of K417N, L452R, E484K and would strengthen the infectivity of the emerging SARS-CoV-2 variants. With the rise of Omicron, some of the spike protein mutations R158G, A262S, S494P, S1242I were reversed; however, the overall shift in mutations boosted the viral infectivity and dropped the virulence factor (Bhattacharyya and Hanage, 2022). Spike R158G was earlier reported to provide fitness to Delta over Alpha variants (Liu et al., 2021) and significantly increased antibody escaping and has been linked with higher infectivity.

Although spike protein constitutes nearly 25% of unique mutations, recent findings have suggested that mutations in the N protein could also alter the function and fitness of SARS-CoV-2 genomes (Rahman et al., 2021; Wu H. et al., 2021). We found that NSP-A394V was associated with nearly 43% breakthrough cases as mentioned earlier, whereas NSP3-P1469S, NSP6-T77A, NSP3-M951I, NS7a-V82A, N-D63G, NS8-E19Stop, N-L139F, NS3-K67N, NSP3-T749A, N-S235F, N-R203K, N-G204R were also annotated in significant frequencies (nearly 5 to 30% cases). Ligand binding, viral oligomerization and packaging, fusion and antibody sensitivity have been reported to be disturbed by these mutations (Arya et al., 2021; Ahamad et al., 2022). Moreover, our findings stand in good agreement with the proposal that in addition to the D614G substitution, mutations in the N protein (R203K/G204R mutations) affect infectivity and virulence of SARS-CoV-2 (Wu S. et al., 2021; Yavarian et al., 2022). Therefore, future vaccine development programs may also focus on regions other than the Spike protein.

### 4.3. Omicron acquired higher infectivity and replaced delta

Spike protein in 21 l had 37 mutations in comparison to 24 mutations in the 21A variant, which significantly changed its binding affinity with the host receptor protein. Binding affinity measured by Gibb’s free energy has been used as a key to infectivity. We found that the binding affinity was similar in 21A Delta and 21 l Omicron (Supplementary Table S1), but a lower dissociation value of 21 L in Omicron suggested a higher stability of receptor-ligand moiety and subsequent faster spread of the variant (Gupta, 2021; Mlcochova et al., 2021). On the other hand, 21 K Omicron has shown relatively higher mean value of –ΔG and Kd than 21 L Omicron and 21A Delta, conferring lower infectivity to the variant. Similarly, Ramachandran plot of the ACE2 receptor -21 K ligand complex suggested several amino acids in the disallowed region, confirming lesser stability than Delta and Omicron 21 L (Figure 6). Moreover, in 21 K, a higher number of proline residues were present in the outer region of generously allowed region, offering lesser permeability and binding with the receptor (Shastri et al., 2021). Further, only five amino acids made significant contact with the receptor motif in comparison with 17 and 18 residues making contact in the cases of 21 L and Delta, respectively, making the binding in 21 K more fragile. Furthermore, Pymol and PdbSum showed relatively longer distance between 21 K-ACE2 in comparison with 21 L and Delta. The absence of salt bridges in 21 K also delimited the interaction capability (Malladi et al., 2021). Since embedding of the salt bridges in the hydrophobic environment stimulates the virus binding energy due to the lowering of dielectric constant; their absence could significantly reduce the affinity in 21 K (Mlcochova et al., 2021). Such a unique combination of mutations in Omicron might have arisen from a recombination between multiple active or dormant variants in the host (Ou et al., 2022). The mutation driven shift in the binding affinity served to confer fitness to Omicron to replace Delta.

## 5. Limitations

A major limitation of this study was a small overall sample size in general and a very small unvaccinated group in the second wave in particular. For statistical comparisons with high confidence at the population level, we would need a sample size way above the one used in this study. Therefore, the statistical comparisons between the waves, and the vaccinated versus unvaccinated groups must be taken with caution. These findings, though partly replicated by a few concurrent and previous publications, should be subjected to further investigations using a much bigger sample size. The other limitation was the lack of full genome coverage in sequencing, which could mask certain interesting mutations, which might be significant in deciding the course of evolution of the SARS-CoV-2 genome.

## 6. Conclusion

The present study aimed at addressing the evolutionary dynamics and mutational profile of SARS-CoV-2 in Uttar Pradesh, India during the 2020–2022 period. One of the key mutations during the early spread of SARS-CoV-2 in Uttar Pradesh, India, was D614G, which was critical in providing infectivity to the virus. This mutation has stayed even in the Delta and Omicron variants, suggesting its critical role in infectivity (Hacisuleyman et al., 2021). In the due course of time, signature combinations of Spike mutations, namely, E156G, F157Del, L452R, T478K, D614G and clustering of NSP-A394V with Spike mutations E156G and R158Del were predominantly associated with vaccination breach infections during the second wave in Uttar Pradesh, India. The key determinants of vaccination breach (F157del, R158del, L452R, T478K, D614G, P681R) were succeeded in the Omicron genome, although some mutations observed in Delta (S1242I, A262S, S494P, R158G) were not seen in the Omicron genome. With this unique selective combination of mutations, Omicron lost virulence and gained infectivity, leading to faster infections but milder effects. Further, the phylogeny tree analysis suggested that the Delta lineage in Uttar Pradesh could arise from the ancestral lineages, but Omicron probably did not directly originate from any of the previously existent single variant, instead it might have arisen from a cryptic genomic architecture involving unusual recombination history. This evolution suggests that new mutations arising in the SARS-CoV-2 genome account for increase in infectivity and reduction in disease severity, eventually leading to the replacement of Delta with Omicron. Significant vaccination breach and wide variations in the infectivity and virulence with molecular changes in the SARS-CoV-2 genome suggest that the emergence of new variants can have significant implications in future pandemics and vaccine efficacy.

## Data availability statement

The data presented in the study are deposited in the GISAID repository at https://gisaid.org under accession heads Asia/India/Uttar Pradesh/CDRI with multiple accession numbers. The accession numbers can be found in the Supplementary material.

## Ethics statement

Ethical review and approval were not required for the study on human participants in accordance with the local legislation and institutional requirements. The patients/participants provided their written informed consent to participate in this study.

## Author contributions

SP, PM, and AA undertook library preparation and sequencing work. AP, RT, PY, UdG, and UjG undertook sample collection and RNA isolation. UjG, UdG, RR, TK, and SR planned the study and arranged funding. RP undertook phylogenetic analysis and provided intellectual inputs. SP undertook structure–function and phylogenetic analysis. SP, PM, and SR wrote the article. All authors contributed to the article and approved the submitted version.

## Funding

The funding for this work was provided by the Council of Scientific and Industrial Research (MLP2021), and the Health Department of Government of Uttar Pradesh (GAP0375). Poonam Mehta would like to thank the University Grants Commission for graduate fellowship (460/CSIR-UGC NET DEC.2017).

## Conflict of interest

The authors declare that the research was conducted in the absence of any commercial or financial relationships that could be construed as a potential conflict of interest.

## Publisher’s note

All claims expressed in this article are solely those of the authors and do not necessarily represent those of their affiliated organizations, or those of the publisher, the editors and the reviewers. Any product that may be evaluated in this article, or claim that may be made by its manufacturer, is not guaranteed or endorsed by the publisher.

## Footnotes

^1^ https://github.com/artic-network/artic-ncov2019

^2^ https://proksee.ca/

^3^ https://wenmr.science.uu.nl/haddock2.4/

^4^ http://cadd.zju.edu.cn/hawkdock/

^5^ https://wenmr.science.uu.nl/prodigy/
