# Supplementary material for: Molecular determinants associated with temporal succession of SARS-CoV-2 variants in Uttar Pradesh, India
Source: Front Microbiol. 2023 Feb 1;14:986729. doi: 10.3389/fmicb.2023.986729 (PMC9929466; doi:10.3389/fmicb.2023.986729)
Supplement: Supplementary file 1 [file Data_Sheet_1.pdf]

Supplementary Tables

Table S1: Binding affinity details: Gibbs free energy and dissociation constant for 21A Delta, 21K Omicron and 21L Omicron in Uttar Pradesh, India during the second and third waves.

Spike_ACE 2 complex	ΔG (kcal mol-1)	Kd (M) at 25.0 ℃	ICs charged-charged	ICs charged-polar	ICs charged-apolar	ICs polar-polar	ICs polar-apolar	ICs apolar-apolar	NIS charged:	NIS apolar:
**Delta (21A)**	-10.18±0.932	7.75E-08±7.19E-08	9.4±4.744	10.1±3.130	17.3±3.880	3.7±2.830	13.7±5.381	12.3±5.451	21.20%±0.022	41.928±0.084%
**21K Omicron**	-8.95±0.812	6.42E-07±8.67E-07	2.9±1.333	2.4±1.231	14±4.400	0.2±0.615	9.5±3.531	16.7±3.672	21.58%±6.640	41.79±0.0674%
**21L Omicron**	-10.68±1.303	1.04E-07±1.7E-07	4.7±3.614	8.5±3.379	19.4±3.816	5.7±3.70	18.3±4.426	17.8±9.185	21.413%±0.028	41.36111±12.731%

Table S2: Details of the binding energy and amino acids involved in *in silico* docking of 21A Delta, 21K Omicron and 21K Omicron Spike with host ACE2.

Spike_ACE2 complex	Binding Free energy	Amino acids falling in Favourable and allowed region	Amino acid falling in disallowed region	Contacts and interface area
**Delta (21A)**	-20.2367±1.118	98.3	1.7%	17 amino acids of Delta spike with 18 aminos acid of ACE2 chain, covering 1072Å^{2}X1068 Å^2, involving 3 salt bridges, 6 hydrogen bonds and 125 non bonded contacts
**21K Omicron**	-13.03±2.196	97.4	2.6%	5 amino acids of 21K Spike with 17 amino acids of ACE2 chain, covering 524Å^{2}X667 Å^2, involving 0 salt bridges, 3 hydrogen bonds and 145 non bonded contacts
**21L Omicron**	-61.8833±2.254	98.3	1.7%	13 amino acids of 21L Spike with 17 amino acids of ACE2 chain, covering 890Å^{2}X951 Å^2, involving 5 salt bridges, 3 hydrogen bonds and 103 non bonded contacts

The data presented in this study are deposited in the GISAID repository under classification 'Asia/India/Uttar Pradesh/CDRI' with the following accession numbers;

For1st wave: (released on 2nd August 2022)
EPI_ISL_14192906, EPI_ISL_14192907, EPI_ISL_14192904, EPI_ISL_14192905, EPI_ISL_14192869, EPI_ISL_14192902, EPI_ISL_14192903, EPI_ISL_14192867, EPI_ISL_14192900, EPI_ISL_14192868, EPI_ISL_14192901, EPI_ISL_14192908, EPI_ISL_14192909, EPI_ISL_14192865, EPI_ISL_14192866, EPI_ISL_14192863, EPI_ISL_14192864, EPI_ISL_14192861, EPI_ISL_14192862, EPI_ISL_14192860, EPI_ISL_14192858, EPI_ISL_14192935, EPI_ISL_14192859, EPI_ISL_14192936,

EPI_ISL_14192856, EPI_ISL_14192933, EPI_ISL_14192857, EPI_ISL_14192934, EPI_ISL_14192890, EPI_ISL_14192891, EPI_ISL_14192854, EPI_ISL_14192898, EPI_ISL_14192931, EPI_ISL_14192855, EPI_ISL_14192899, EPI_ISL_14192932, EPI_ISL_14192852, EPI_ISL_14192896, EPI_ISL_14192853, EPI_ISL_14192897, EPI_ISL_14192930, EPI_ISL_14192850, EPI_ISL_14192894, EPI_ISL_14192851, EPI_ISL_14192895, EPI_ISL_14192892, EPI_ISL_14192893, EPI_ISL_14192928, EPI_ISL_14192929, EPI_ISL_14192926, EPI_ISL_14192927, EPI_ISL_14192924, EPI_ISL_14192925, EPI_ISL_14192889, EPI_ISL_14192922, EPI_ISL_14192923, EPI_ISL_14192880, EPI_ISL_14192887, EPI_ISL_14192920, EPI_ISL_14192888, EPI_ISL_14192921, EPI_ISL_14192885, EPI_ISL_14192886, EPI_ISL_14192883, EPI_ISL_14192884, EPI_ISL_14192881, EPI_ISL_14192882, EPI_ISL_14192917, EPI_ISL_14192918, EPI_ISL_14192915, EPI_ISL_14192916, EPI_ISL_14192913, EPI_ISL_14192914, EPI_ISL_14192878, EPI_ISL_14192911, EPI_ISL_14192879, EPI_ISL_14192912, EPI_ISL_14192919, EPI_ISL_14192876, EPI_ISL_14192877, EPI_ISL_14192910, EPI_ISL_14192874, EPI_ISL_14192875, EPI_ISL_14192872, EPI_ISL_14192873, EPI_ISL_14192870, EPI_ISL_14192871

For 2nd wave: (released on 3rd august)
EPI_ISL_14211696, EPI_ISL_14211697, EPI_ISL_14211694, EPI_ISL_14211695, EPI_ISL_14211698, EPI_ISL_14211699, EPI_ISL_14211809, EPI_ISL_14211803, EPI_ISL_14211804, EPI_ISL_14211801, EPI_ISL_14211802, EPI_ISL_14211807, EPI_ISL_14211808, EPI_ISL_14211805, EPI_ISL_14211806, EPI_ISL_14211800, EPI_ISL_14211670, EPI_ISL_14211791, EPI_ISL_14211671, EPI_ISL_14211792, EPI_ISL_14211790, EPI_ISL_14211669, EPI_ISL_14211663, EPI_ISL_14211784, EPI_ISL_14211664, EPI_ISL_14211785, EPI_ISL_14211661, EPI_ISL_14211782, EPI_ISL_14211662, EPI_ISL_14211783, EPI_ISL_14211788, EPI_ISL_14211667, EPI_ISL_14211789, EPI_ISL_14211668, EPI_ISL_14211665, EPI_ISL_14211786, EPI_ISL_14211787, EPI_ISL_14211666, EPI_ISL_14211780, EPI_ISL_14211660, EPI_ISL_14211781, EPI_ISL_14211779, EPI_ISL_14211658, EPI_ISL_14211659, EPI_ISL_14211652, EPI_ISL_14211773, EPI_ISL_14211653, EPI_ISL_14211774, EPI_ISL_14211650, EPI_ISL_14211771, EPI_ISL_14211651, EPI_ISL_14211772, EPI_ISL_14211777, EPI_ISL_14211656, EPI_ISL_14211778, EPI_ISL_14211657, EPI_ISL_14211654, EPI_ISL_14211775, EPI_ISL_14211655, EPI_ISL_14211776, EPI_ISL_14211692, EPI_ISL_14211693, EPI_ISL_14211690, EPI_ISL_14211691, EPI_ISL_14211685, EPI_ISL_14211686, EPI_ISL_14211683, EPI_ISL_14211684, EPI_ISL_14211689, EPI_ISL_14211687, EPI_ISL_14211688, EPI_ISL_14211681, EPI_ISL_14211682, EPI_ISL_14211680, EPI_ISL_14211674, EPI_ISL_14211795, EPI_ISL_14211675, EPI_ISL_14211796, EPI_ISL_14211672, EPI_ISL_14211793, EPI_ISL_14211673, EPI_ISL_14211794, EPI_ISL_14211799, EPI_ISL_14211678, EPI_ISL_14211679, EPI_ISL_14211797, EPI_ISL_14211676, EPI_ISL_14211798, EPI_ISL_14211677, EPI_ISL_14211748, EPI_ISL_14211749, EPI_ISL_14211746, EPI_ISL_14211747, EPI_ISL_14211740, EPI_ISL_14211861, EPI_ISL_14211741, EPI_ISL_14211862, EPI_ISL_14211860, EPI_ISL_14211744, EPI_ISL_14211745, EPI_ISL_14211742, EPI_ISL_14211863, EPI_ISL_14211743, EPI_ISL_14211864, EPI_ISL_14211737, EPI_ISL_14211858, EPI_ISL_14211738, EPI_ISL_14211859, EPI_ISL_14211735, EPI_ISL_14211856, EPI_ISL_14211736, EPI_ISL_14211857, EPI_ISL_14211739, EPI_ISL_14211850, EPI_ISL_14211730, EPI_ISL_14211851, EPI_ISL_14211733, EPI_ISL_14211854, EPI_ISL_14211734, EPI_ISL_14211855, EPI_ISL_14211731, EPI_ISL_14211852, EPI_ISL_14211732, EPI_ISL_14211853, EPI_ISL_14211770, EPI_ISL_14211649, EPI_ISL_14211768, EPI_ISL_14211647,

EPI_ISL_14211769, EPI_ISL_14211648, EPI_ISL_14211762, EPI_ISL_14211763, EPI_ISL_14211760, EPI_ISL_14211761, EPI_ISL_14211766, EPI_ISL_14211767, EPI_ISL_14211764, EPI_ISL_14211765, EPI_ISL_14211759, EPI_ISL_14211757, EPI_ISL_14211758, EPI_ISL_14211751, EPI_ISL_14211752, EPI_ISL_14211750, EPI_ISL_14211755, EPI_ISL_14211756, EPI_ISL_14211753, EPI_ISL_14211754, EPI_ISL_14211704, EPI_ISL_14211825, EPI_ISL_14211705, EPI_ISL_14211826, EPI_ISL_14211702, EPI_ISL_14211823, EPI_ISL_14211703, EPI_ISL_14211824, EPI_ISL_14211708, EPI_ISL_14211829, EPI_ISL_14211709, EPI_ISL_14211706, EPI_ISL_14211827, EPI_ISL_14211707, EPI_ISL_14211828, EPI_ISL_14211700, EPI_ISL_14211821, EPI_ISL_14211701, EPI_ISL_14211822, EPI_ISL_14211820, EPI_ISL_14211814, EPI_ISL_14211815, EPI_ISL_14211812, EPI_ISL_14211813, EPI_ISL_14211818, EPI_ISL_14211819, EPI_ISL_14211816, EPI_ISL_14211817, EPI_ISL_14211810, EPI_ISL_14211811, EPI_ISL_14211726, EPI_ISL_14211847, EPI_ISL_14211727, EPI_ISL_14211848, EPI_ISL_14211724, EPI_ISL_14211845, EPI_ISL_14211725, EPI_ISL_14211846, EPI_ISL_14211728, EPI_ISL_14211849, EPI_ISL_14211729, EPI_ISL_14211840, EPI_ISL_14211722, EPI_ISL_14211843, EPI_ISL_14211723, EPI_ISL_14211844, EPI_ISL_14211720, EPI_ISL_14211841, EPI_ISL_14211721, EPI_ISL_14211842, EPI_ISL_14211715, EPI_ISL_14211836, EPI_ISL_14211716, EPI_ISL_14211837, EPI_ISL_14211713, EPI_ISL_14211834, EPI_ISL_14211714, EPI_ISL_14211835, EPI_ISL_14211719, EPI_ISL_14211717, EPI_ISL_14211838, EPI_ISL_14211718, EPI_ISL_14211839, EPI_ISL_14211711, EPI_ISL_14211832, EPI_ISL_14211712, EPI_ISL_14211833, EPI_ISL_14211830, EPI_ISL_14211710, EPI_ISL_14211831

For 3rd wave, released date 4th august

EPI_ISL_14227259, EPI_ISL_14227265, EPI_ISL_14227386, EPI_ISL_14227264, EPI_ISL_14227385, EPI_ISL_14227263, EPI_ISL_14227384, EPI_ISL_14227262, EPI_ISL_14227383, EPI_ISL_14227269, EPI_ISL_14227268, EPI_ISL_14227389, EPI_ISL_14227267, EPI_ISL_14227388, EPI_ISL_14227266, EPI_ISL_14227387, EPI_ISL_14227261, EPI_ISL_14227382, EPI_ISL_14227260, EPI_ISL_14227381, EPI_ISL_14227380, EPI_ISL_14227276, EPI_ISL_14227397, EPI_ISL_14227275, EPI_ISL_14227396, EPI_ISL_14227274, EPI_ISL_14227395, EPI_ISL_14227273, EPI_ISL_14227394, EPI_ISL_14227279, EPI_ISL_14227278, EPI_ISL_14227399, EPI_ISL_14227277, EPI_ISL_14227398, EPI_ISL_14227272, EPI_ISL_14227393, EPI_ISL_14227271, EPI_ISL_14227392, EPI_ISL_14227270, EPI_ISL_14227391, EPI_ISL_14227390, EPI_ISL_14227239, EPI_ISL_14227238, EPI_ISL_14227359, EPI_ISL_14227237, EPI_ISL_14227358, EPI_ISL_14227243, EPI_ISL_14227364, EPI_ISL_14227242, EPI_ISL_14227363, EPI_ISL_14227241, EPI_ISL_14227362, EPI_ISL_14227240, EPI_ISL_14227361, EPI_ISL_14227368, EPI_ISL_14227247, EPI_ISL_14227367, EPI_ISL_14227246, EPI_ISL_14227245, EPI_ISL_14227366, EPI_ISL_14227244, EPI_ISL_14227365, EPI_ISL_14227360, EPI_ISL_14227249, EPI_ISL_14227369, EPI_ISL_14227248, EPI_ISL_14227254, EPI_ISL_14227375, EPI_ISL_14227253, EPI_ISL_14227374, EPI_ISL_14227252, EPI_ISL_14227373, EPI_ISL_14227251, EPI_ISL_14227372, EPI_ISL_14227379, EPI_ISL_14227258, EPI_ISL_14227378, EPI_ISL_14227257, EPI_ISL_14227377, EPI_ISL_14227256, EPI_ISL_14227255, EPI_ISL_14227376, EPI_ISL_14227250, EPI_ISL_14227371, EPI_ISL_14227370, EPI_ISL_14227339, EPI_ISL_14227338, EPI_ISL_14227337, EPI_ISL_14227336, EPI_ISL_14227221, EPI_ISL_14227342, EPI_ISL_14227341, EPI_ISL_14227340, EPI_ISL_14227225, EPI_ISL_14227346, EPI_ISL_14227224,

EPI_ISL_14227345, EPI_ISL_14227223, EPI_ISL_14227344, EPI_ISL_14227222, EPI_ISL_14227343, EPI_ISL_14227229, EPI_ISL_14227228, EPI_ISL_14227349, EPI_ISL_14227227, EPI_ISL_14227348, EPI_ISL_14227226, EPI_ISL_14227347, EPI_ISL_14227232, EPI_ISL_14227353, EPI_ISL_14227231, EPI_ISL_14227352, EPI_ISL_14227230, EPI_ISL_14227351, EPI_ISL_14227350, EPI_ISL_14227236, EPI_ISL_14227357, EPI_ISL_14227235, EPI_ISL_14227356, EPI_ISL_14227234, EPI_ISL_14227355, EPI_ISL_14227233, EPI_ISL_14227354, EPI_ISL_14227317, EPI_ISL_14227438, EPI_ISL_14227316, EPI_ISL_14227437, EPI_ISL_14227315, EPI_ISL_14227436, EPI_ISL_14227314, EPI_ISL_14227435, EPI_ISL_14227319, EPI_ISL_14227318, EPI_ISL_14227439, EPI_ISL_14227320, EPI_ISL_14227441, EPI_ISL_14227440, EPI_ISL_14227324, EPI_ISL_14227445, EPI_ISL_14227323, EPI_ISL_14227444, EPI_ISL_14227322, EPI_ISL_14227443, EPI_ISL_14227321, EPI_ISL_14227442, EPI_ISL_14227328, EPI_ISL_14227449, EPI_ISL_14227327, EPI_ISL_14227448, EPI_ISL_14227326, EPI_ISL_14227447, EPI_ISL_14227325, EPI_ISL_14227446, EPI_ISL_14227329, EPI_ISL_14227331, EPI_ISL_14227452, EPI_ISL_14227330, EPI_ISL_14227451, EPI_ISL_14227450, EPI_ISL_14227335, EPI_ISL_14227334, EPI_ISL_14227333, EPI_ISL_14227332, EPI_ISL_14227416, EPI_ISL_14227415, EPI_ISL_14227414, EPI_ISL_14227413, EPI_ISL_14227419, EPI_ISL_14227418, EPI_ISL_14227417, EPI_ISL_14227302, EPI_ISL_14227423, EPI_ISL_14227301, EPI_ISL_14227422, EPI_ISL_14227300, EPI_ISL_14227421, EPI_ISL_14227420, EPI_ISL_14227306, EPI_ISL_14227427, EPI_ISL_14227305, EPI_ISL_14227426, EPI_ISL_14227304, EPI_ISL_14227425, EPI_ISL_14227303, EPI_ISL_14227424, EPI_ISL_14227309, EPI_ISL_14227308, EPI_ISL_14227429, EPI_ISL_14227307, EPI_ISL_14227428, EPI_ISL_14227430, EPI_ISL_14227313, EPI_ISL_14227434, EPI_ISL_14227312, EPI_ISL_14227433, EPI_ISL_14227311, EPI_ISL_14227432, EPI_ISL_14227310, EPI_ISL_14227431, EPI_ISL_14227401, EPI_ISL_14227400, EPI_ISL_14227405, EPI_ISL_14227404, EPI_ISL_14227403, EPI_ISL_14227402, EPI_ISL_14227409, EPI_ISL_14227408, EPI_ISL_14227407, EPI_ISL_14227406, EPI_ISL_14227412, EPI_ISL_14227411, EPI_ISL_14227410, EPI_ISL_14227287, EPI_ISL_14227286, EPI_ISL_14227285, EPI_ISL_14227284, EPI_ISL_14227289, EPI_ISL_14227288, EPI_ISL_14227283, EPI_ISL_14227282, EPI_ISL_14227281, EPI_ISL_14227280, EPI_ISL_14227298, EPI_ISL_14227297, EPI_ISL_14227296, EPI_ISL_14227295, EPI_ISL_14227299, EPI_ISL_14227290, EPI_ISL_14227294, EPI_ISL_14227293, EPI_ISL_14227292, EPI_ISL_14227291
